# Antibacterial activity and mechanism of luteolin isolated from *Lophatherum gracile* Brongn. against multidrug-resistant *Escherichia coli*

**DOI:** 10.3389/fphar.2024.1430564

**Published:** 2024-06-24

**Authors:** Yahao Ding, Guilan Wen, Xingke Wei, Hao Zhou, Chunjie Li, Zhengqin Luo, Deyuan Ou, Jian Yang, Xuqin Song

**Affiliations:** ^1^ Laboratory of Animal Genetics, Breeding and Reproduction in the Plateau Mountainous Region, Ministry of Education, Guizhou University, Guiyang, China; ^2^ College of Animal Science, Guizhou University, Guiyang, China; ^3^ Pearl River Fisheries Research Institute, Chinese Academy of Fishery Sciences, Guangzhou, China; ^4^ Laboratory of Pulmonary Immunology and Inflammation, Frontiers Science Center for Disease-related Molecular Network, Sichuan University, Chengdu, China

**Keywords:** *Lophatherum gracile* Brongn., luteolin, multidrug-resistant *Escherichia coli*, antibacterial mechanism, untargeted metabolomics

## Abstract

Infections caused by multidrug-resistant (MDR) bacteria have become a major challenge for global healthcare systems. The search for antibacterial compounds from plants has received increasing attention in the fight against MDR bacteria. As a medicinal and edible plant, *Lophatherum gracile* Brongn. (*L. gracile*) has favorable antibacterial effect. However, the main antibacterial active compound and its antimicrobial mechanism are not clear. Here, our study first identified the key active compound from *L. gracile* as luteolin. Meanwhile, the antibacterial effect of luteolin was detected by using the broth microdilution method and time-kill curve analysis. Luteolin can also cause morphological structure degeneration and content leakage, cell wall/membrane damage, ATP synthesis reduction, and downregulation of mRNA expression levels of sulfonamide and quinolones resistance genes in multidrug-resistant *Escherichia coli* (MDR *E. coli*). Furthermore, untargeted UPLC/Q-TOF-MS-based metabolomics analysis of the bacterial metabolites revealed that luteolin significantly changed riboflavin energy metabolism, bacterial chemotaxis cell process and glycerophospholipid metabolism of MDR *E. coli*. This study suggests that luteolin could be a potential new food additive or preservative for controlling MDR *E. coli* infection and spread.

## 1 Introduction

*Escherichia coli* (*E. coli*) is a Gram-negative facultative anaerobe, which is the main cause of foodborne infection and can cause gastrointestinal and parenteral diseases, such as diarrhea, enteritis, bacteremia, urinary tract infection, etc (Rahman et al., 2020; Xu et al., 2022). More worryingly, due to the extensive use and abuse of antibiotics, the drug resistance of human and animal-derived *E. coli* has increased dramatically, which has already posed a major threat to global public health and food safety. At present, *E. coli* has developed resistance to a variety of antibiotics, such as polymyxins and quinolones, resulting in multidrug-resistant *E. coli* (MDR *E. coli*) and even super-drug resistant bacteria. In addition, according to statistics the formation of biofilms of drug-resistant bacteria plays a role in 80% of clinical infections (Cui et al., 2022). More shockingly, the drug resistance of bacteria is closely related to the formation of biofilms. The stronger the drug resistance of bacteria, the denser the biofilm formed (Bhardwaj et al., 2021), which further increases the difficulty of antibiotic treatment. Therefore, to reduce the widespread use of antibiotics and reduce the impact of drug-resistant *E. coli*, many researchers have focused on the development of natural botanicals from plant extracts. However, there are few studies on the antibacterial effect of Gram-negative bacteria, especially foodborne *E. coli* with the most serious drug resistance.

*Lophatherum gracile* Brongn. (*L. gracile*), an excellent gramineous plant that has the function of both medicine and foodstuff in China and around the world, is the dry stem and leaf of *L. gracile* (Li et al., 2024). This herb has a variety of pharmacological effects, such as anti-bacterial, anti-viral, anti-oxidant, anti-inflammatory and hyperglycemic (Lai et al., 2021). Recently, besides being used as a traditional medicine, the application of *L. gracile* in food and related fields is also increasing. For example, *L. gracile* can not only be used in combination with Chinese herbal medicines such as lotus leaves to make a functional beverage, but also can be used as an auxiliary material for alcoholic beverages such as Zhuyeqing liquor to play a certain role in liver protection, immune protection and other healthcare (Gao et al., 2014; Gao et al., 2015). Moreover, *L. gracile* could also commonly used as tea in the folk. A chitosan film containing *L. gracile* extract (5%) was developed, compared with ordinary chitosan film, the preservation time of fried bighead carp was greatly prolonged and the preservation effect was remarkable (Chen et al., 2020). *L. gracile* is rich in natural flavonoids and polysaccharides. Related literature have verified that a series of flavonoids can be isolated from *L. gracile* by UPLC-tography fingerprints or LC-MS/MS, etc., of which isoorientin, orientin, rutin, vitexin, isovitexin, quercetin and luteolin are the main pharmacological active ingredients (Fu et al., 2014; Li et al., 2021; Liu et al., 2022). It has been reported that the ethanol extract of *L. gracile* and its flavonoid glycosides have proved to have good antibacterial effects (Adamczak et al., 2019; Chen et al., 2019). However, the antibacterial active ingredients and mechanism of *L. gracile* are still unclear.

Although the mechanism of bacterial resistance is difficult to decipher, in addition to conventional mechanism research, it is a new choice to explain the molecular mechanism of drug resistance at metabolic level (Li et al., 2015). Compared with small changes in gene and protein levels, metabolomics can scale up research through quantitative detection of existing metabolites to more accurately and directly reflect the terminal and phenotypic information of living organisms, then potentially promote the discovery of new alternative drug targets. For the study of bacterial resistance, the commonly used analysis method is untargeted metabolomics, which can quantitatively analyze all metabolites in the organism to fully amplify the mechanism of action of antibacterial drugs and the recognition and identification of potential drug targets in the process of drug development (Hoerr et al., 2016; Han et al., 2018). Moreover, the detection methods are mostly high-performance liquid chromatography tandem mass spectrometry (LC-MS/MS), which could detect as more metabolites as possible at a very low detection limit, further to combine high-throughput analytical chemistry and multivariate data analysis to characterize the metabolite phenotype and deepen the understanding of the antibacterial mechanism (Fortuin and Soares, 2022) Most bacteria rely on metabolism for growth and reproduction. Changes in the concentration of one or more metabolites could reflect changes in intracellular enzyme activity, absorption and consumption levels, and excretion rates. This is also a response to changes in external conditions (Rinschen et al., 2019). It has been reported that the same antibiotics have different effects on the metabolic level of methicillin-sensitive and methicillin-insensitive *Staphylococcus aureus* (Schelli et al., 2017), indicating that there may be different stress response mechanisms or metabolic pathways in the metabolic process of the two strains. Therefore, compared with the study of metabolomics of sensitive strains, the study of drug-resistant bacterial infections that are difficult to treat could find more significant changes in key metabolic pathways, thus providing more therapeutic approaches and more innovative significance. More interestingly, studies have shown that the anti-obesity effect of quercetin may be achieved by regulating the abundance of gut microbiota induced by high-fat diet and promoting the unbalanced death of harmful bacterial metabolic pathways (Su et al., 2022). However, natural botanicals (such as luteolin) with multiple targets have rarely been reported to analyze the antibacterial mechanism from the perspective of untargeted metabolomics. Metabolomics research based on previous antibacterial drugs has brought rich analytical experience (Zhang and Zhang, 2020; Leng et al., 2021), which provides great convenience for us to further explore the molecular mechanism of luteolin against MDR *E. coli*.

The purpose of this study was to obtain the best antibacterial ingredient in *L. gracile* and to reveal its antimicrobial mechanism on MDR *E. coli*. After screening the antibacterial active components of flavonoids from *L. gracile*, luteolin was deemed the best candidate for further study. We investigated the dynamic antibacterial effect of luteolin on MDR *E. coli* growth curve. Moreover, we conducted measurements of cell wall and cell membrane damage, ATP synthesis, cell death and viability, bacterial morphology and drug resistance gene expression caused by luteolin. Furthermore, untargeted metabolomics is of great significance for understanding the antibacterial molecular mechanism of luteolin. This study will help to provide new modes of action or new antibacterial targets against MDR bacteria, which is expected to provide a theoretical basis for the development of natural antibacterial agents or food preservative.

## 2 Material and methods

### 2.1 Chemicals and reagents

Eosin-methylene blue agar base (EMB) and Mueller-Hinton broth (MHB) were purchased from Beijing Aoboxing Bio-Technology Co., ltd. (Beijing, China). Luteolin, quercetin, rutin, orientin, isoorientin, vitexin, isovitexin and dimethyl sulfoxide (DMSO) were purchased from Yuanye Bio-Technology Co., Ltd. (Shanghai, China), both with a purity of ≥98%. Polymyxin B sulfate (>6,500 IU/mg, USP) was purchased from Dalian Meilun Biotechnology Co., Ltd (Tianjin, China). Tween-80 (Oleic acid approx.70%) was purchased from Beijing Solarbio Science & Technology Co., Ltd. (Beijing, China). Ethanol absolute, petroleum ether (60–90°C), ethyl acetate (EtOAc), n-butanol and dichloromethane were purchased from Tianjin Kemiou Chemical Reagent Factory (Tianjin, China), both with a purity of ≥99.5%. The HPLC-grade solvents of formic acid, acetonitrile (ACN) and methanol (MeOH) were from Fisher Scientific (Fairlawn, NJ, United States of America).

### 2.2 Bacterial strains

The tested MDR *E. coli* strain (GZGY201912) was kindly provided by Professor Guilan Wen and isolated from broilers. The test strains carried six resistance genes (*sul2*, *sul3*, *gyrA*, *gyrB*, *oqxA* and *parC*). The quality control strain of *E. coli* (ATCC25922) was purchased from Beijing Microbiological Culture Collection Center. (Beijing, China). The MDR *E. coli* was incubated on EMB plates and maintained in an electrothermal incubator at 37°C for 16 h. A single colony was picked from the EMB plate and inoculated into MHB medium, and then incubated in a 37°C constant temperature water bath shaker for 16 h. Finally, the cultured bacterial solution was diluted to 1×10^6^ colony forming units per milliliter (CFU/mL) for subsequent experiments. All strains were cultured according to the Clinical and Laboratory Standards Institute (CLSI) guidelines.

### 2.3 Plant material and preparation of *L. gracile* extract

The *L. gracile* (Batch No. 20220201) used in this study were purchased from Jirentang Chinese Herbal Pieces Factory, Guiyang City. 100 g of dried *L. gracile* was crushed and soaked in 1 L of 95% ethanol for 12 h and ultrasonically extracted at 45°C for 3 times, 1 h each time. After the filtration of the supernatant, a 65°C rotary evaporator was used to concentrate to obtain the crude extract (6.5 g). The extract was dried to constant weight in an oven at 60°C. Then the extract was uniformly dispersed in 200 mL distilled water, and successively extracted with 100 mL of petroleum ether, EtOAc, dichloromethane and n-butanol for 4 times, 1 h each time. Finally, the above extract of each solvent was concentrated, and dissolved in a mixture of 3% Tween-80 and DMSO to different concentrations of 102.4 mg/mL, 25.6 mg/mL, 51.2 mg/mL, 51.2 mg/mL. The biological activity of each extract against the MDR *E. coli* was preliminarily determined by microbroth dilution method, and the results showed that EtOAc extract had the best anti-MDR *E. coli* activity. Therefore, in order to find compounds against the MDR *E. coli*, the EtOAc extract was analyzed using liquid chromatography with tandem mass spectrometry (LC-MS/MS).

### 2.4 Identification of compounds from EtOAc extract by LC-MS/MS

The main pharmacological active components of flavonoids in *L. gracile* are isoorientin, orientin, rutin, vitexin, isovitexin, quercetin and luteolin, respectively. Therefore, this study used standard substances to identify and quantitatively analyze the EtOAc fraction. The LC separation was performed in an HPLC 1290 series with a ZORBAX SB-C_18_ column (2.1 mm × 100 mm i.d., 1.8 µm particle size, Agilent Technologies^®^, Santa Clara, CA, United States of America). The mobile phase consisted of methanol (A) and 0.5% acetic acid in water solution (B) with the following gradient elution program: 90% A, 0–1 min; 90%–85% A, 1–2 min; 85%–65% A, 2–5 min; 65%–30% A, 5–5.1 min; 30%–20% A, 5.1–9 min; 20%–90% A, 9–10 min. The total run time was 10 min with a constant flow rate of 0.3 mL/min. The injection volume was 10 μL. MS analysis was performed on an Agilent system 6470 series triple-quadrupole mass spectrometer using multiple-reaction monitoring (MRM) transition under electrospray negative ion (ESI^−^) mode. The operation conditions were as follows: capillary voltage, 3.5 kV; nebulizer gas, 40 psi; sheath gas temperature, 350°C; sheath gas flow rate, 11 L/min; ionization gas flow rate, 8 L/min. Mass spectral parameters for the seven target analytes are shown in Supplementary Table S1.

### 2.5 Susceptibility assays

The minimum inhibitory concentration (MICs) and minimum bactericidal concentration (MBCs) of isolated compounds were determined by the microbroth dilution method. The maximum dissolved concentration of the isolated compound was dissolved in 3% DMSO, and 2-fold serially diluted in 96-well plates using MHB containing the bacterial solution to a final bacterial concentration of 1 × 10^6^ CFU/mL. The prepared 96-well plates were incubated in the incubator at 37°C for 16 h, and the bacterial growth and turbidity of each well were observed. The MBC was determined to be the lowest concentration when the sterile colony grew. The positive control (polymyxin E sulfate, 16 μg/mL), sterile control and MHB containing 3% DMSO were used as blank controls. All experiments were repeated three times.

### 2.6 *In vitro* antibacterial activity of luteolin against MDR *Escherichia coli*

#### 2.6.1 Time-kill assay

Time-kill curves are mainly used to monitor the growth and death of bacteria under different drug concentrations and are widely used to evaluate the drug-time relationship. The prepared 1 × 10^6^ CFU/mL MDR *E. coli* bacterial suspension and different concentrations of luteolin were added to six 50 mL sterile polypropylene centrifuge tubes, respectively, with a total volume of 30 mL. Finally, the dissolution concentration of luteolin was maintained at 1/4 × MIC, 1/2 × MIC, 1 × MIC, and 2 × MIC, respectively. Meanwhile, no drug was added as the positive growth control group, and 3% DMSO was used as the negative control group. The above six groups of centrifuge tubes were placed in a 37°C constant temperature oscillator at 180 r/min for 24 h. 1 mL of samples were taken at 0, 2, 4, 6, 8, 10, 12, 24 h and the optical density at 600 nm was measured by microplate reader (Synergy H1, Diege Bio-Science & Technology Shanghai Co., China).

#### 2.6.2 Leakage of intracellular content

Alkaline phosphatase (AKP) is a protease that exists between the cell membrane and cell wall, and can only leak out when the cell wall is damaged. Therefore, AKP activity was used to evaluate the degree of cell wall damage (Yang et al., 2020). The MDR *E. coli* (10^6^ CFU/mL) was treated with different concentrations of luteolin, and a blank growth group was set up to explore the damage degree of the bacterial cell wall. It was placed in a 37°C constant temperature oscillator at 180 r/min for 24 h. The bacterial solution of 0, 4, 8, and 12 h was centrifuged at 4,000 *g* for 10 min and the supernatant was taken to another 2 mL sterile centrifuge tube. The leakage of AKP after treatment with different concentrations of luteolin was detected by an AKP kit (Jiancheng Bioengineering Institute, Nanjing, China) and a microplate reader at 520 nm.

#### 2.6.3 Inhibition of biofilm formation

Based on a previous study (Deng et al., 2019), the biofilm formation ability of MDR *E. coli* was evaluated by micro-enzyme labeling method. In brief, 100 μL (approximately 10^6^ CFU/mL) of bacterial solution diluted with MHB medium was added to each 96-well cell culture plate, and different concentrations of luteolin was added to evaluate the degree of biofilm growth inhibition. After static culture at 37°C for 36 h, the culture medium was discarded and gently washed 3 times with 200 μL 10 mM sterile phosphate-buffered saline (PBS; pH = 7.2). 100 μL of 95% methanol was added to each well and fixed for 15 min. The liquid was discarded and the well was naturally dried. The wells were stained with 100 μL of 0.1% (w/v) crystal violet for 30 min, and washed with PBS 3 times. Residual crystal violet was dissolved in 200 μL of 95% ethanol and incubated at 37°C for 5 min. Finally, the OD value of each well was measured by the microplate reader at 595 nm.

#### 2.6.4 Determination of ATP concentration

The diluted drug-resistant test bacterial suspension (∼10^7^ CFU/mL) was centrifuged at 4°C, 8,000×g for 10 min, and the cells were collected to obtain a precipitate. After washing with PBS, the cells were resuspended in an equal volume of MH broth medium. The bacterial suspension was treated with different concentrations of luteolin for 3 h, and the bacteria were collected again after centrifugation. At the same time, a blank growth control group was set up. The weight of the bacteria was suspended in PBS, and the absorbance at 660 nm of the OD value was measured by the Na^+^K^+^-ATPase activity detection kit (Solarbio Technology Co., Ltd., Beijing, China) to calculate the change of ATP content in the bacterial cells.

#### 2.6.5 Scanning electron microscopy (SEM) and transmission electron microscopy (TEM)

The changes in morphology and ultrastructure before and after the interaction between luteolin and MDR *E. coli* were observed using SEM and TEM. The tested MDR *E. coli* (approximately 10^6^ CFU/mL) was treated with different concentrations of luteolin (1/2 × MIC, 1 × MIC, 2 × MIC) or without luteolin and for 16 h at 37°C under 180 r/min. The treated bacterial solution was centrifuged and fixed with 2.5% glutaraldehyde at 4°C for 4 h. Then, the bacteria solution was dehydrated with graded concentrations of ethanol and immersed in isoamyl acetate for 15 min. After drying, samples were sputtered with a gold layer and observed using a Hitachi-SU8100 SEM (Hitachi, Tokyo, Japan). As for TEM characterization, samples treated with different concentrations of luteolin (1 × MIC, 2 × MIC) were prepared using the embedding method and resin blocks were cut to 60–80 nm lamina on the Leica UC7 ultra-microtome (Leica, Germany). Finally, TEM characterization was carried out by a Hitachi-HT7800 TEM (Hitachi, Tokyo, Japan).

### 2.7 Bacterial viability assay

The viability of the MDR *E. coli* in the medium was studied by green nucleic acid (SYTO 9) and propidium iodide (PI) double dye fluorescence staining, and the operation was carried out concerning the LIVE/DEAD Bacterial Double Stain Kit (Maokang Bio-Science & Technology Co., Shanghai, China). The exponential growth phase of the bacterial suspension (∼10^6^ CFU/mL) was treated with different concentrations of luteolin for 16 h at 37°C under 180 r/min. After centrifugation at 10,000 *g* for 10 min, bacteria were resuspended with 0.85% sterile saline and incubated at room temperature for 1 h. 1 mL bacterial suspension was added with 3 μL SYTO 9 (2.5 μM) and PI (15 μM) dye pre-mixed solution for incubation at room temperature for 15 min in the dark. After 5 μL of dyed bacterial suspension was dried, the anti-fluorescence quencher was added. Finally, the viable cells (green) and dead cells (red) were observed using NIKON Eclipse Ti confocal laser-scanning (CLSM, Nikon, Tokyo, Japan).

### 2.8 Quantitative reverse-transcription PCR

The bacterial cultures (∼10^6^ CFU/mL) were treated with luteolin and a blank growth control group was established for 16 h at 37°C under 180 r/min. The total RNA was extracted by centrifugal column bacterial RNA extraction kit (Tiangen Biotech Co., Beijing, China). Total RNA was reverse transcribed into cDNA using HyperScript™ RT SuperMix (APE×BIO Technology LLC, Houston, America) Quantitative PCR primers were designed according to the drug-resistance gene sequence on the plasmid of the test strain, and 16S rRNA was used as the internal reference gene. The primer sequence is shown in Supplementary Table S2. Quantitative real-time PCR (qRT-PCR) amplification was performed using the HotStart™ SYBR Green PCR Kit and the CFX Connect Real-Time PCR system (Bio-Rad, Hercules, America) under the following conditions; an initial denaturation step of 2 min at 95°C followed by 40 cycles of 95°C denaturation for 15 s and 60°C annealing for 30 s. Finally, the relative gene expression was calculated by the 2^−ΔΔCT^ method.

### 2.9 Metabolomics analysis

Further to more intuitively and effectively reflect the mechanism of life activities involved in the MDR *E. coli*. The potential effects of luteolin on metabolites and metabolic pathways of the MDR *E. coli* were studied by untargeted metabolomics. The MDR *E. coli* (approximately 10^6^ CFU/mL) was treated with 1/4 × MIC concentration of luteolin for 10 h at 37°C, 180 r/min. The bacterial solutions were collected in a 1.5 mL sterile centrifuge tube, centrifuged at 4°C, 4,000 g for 3 min, removed the supernatant, washed the bacteria twice with pre-cooled PBS, and repeated centrifugation to collect 100 mg of bacterial precipitate. Finally, it was quickly frozen in liquid nitrogen and stored at-80°C for testing (Winder et al., 2008). The bacterial precipitate to be tested was transferred from the refrigerator at −80°C to −4°C and slowly thawed. The pre-cooled ACN/MeOH solution (v:v = 1:1) was added to the tube. The samples were shaken with a vortex shaker for 30 s, followed by ultrasonic extraction in a water bath for 10 min. After standing at −20°C for 1 h, the sample was centrifuged at 4°C, 13,000 r/min for 15 min and the supernatant was taken and freeze-dried. The precipitate was redissolved with 50% ACN in water, and the extraction procedure was repeated. The supernatant was filtered (0.22 μm) into the injection bottle for LC-MS/MS analysis. In addition, 10 μL of each sample was mixed to make a quality control (QC) sample to be tested. The instrumentation was based on an ultra-high performance liquid chromatography system (Shimadzu 30 series, Japan) combined with Quadrupole Time-of-Flight mass spectrometry system (SCIEX, Triple TOF 5600+ series, Framingham, MA, United States of America, UPLC/Q-TOF-MS). Pre-separation was carried out on an ACQUITY-UPLC-T3 column (100 mm*2.1 mm, 1.7 µm, Waters, United Kingdom). The column temperature was kept at 50°C. The flow rate was 0.3 mL/min and the injection volume of the single needle was 2 μL. The mobile phase consisted of 0.1% FA in ACN (A) and 0.1% FA (B) with the following gradient elution program: 5% A, 0–0.5 min; 5%–70% A, 0.5–2.5 min; 70%–100% A, 2.5–7.5 min; 100% A, 7.5–9.0 min; 100%–5% A, 9.0–9.5 min; 5% A, 9.0–9.5 min. The detailed parameters are as follows: Ion Source Gas1: 50 psi, Ion Source Gas2: 50 psi, Curtain Gas: 35 psi, Source Temperature: 500°C, IonSapary Voltage Floating: 5500 V and-4500 V (positive & negative); Declustering Potential (DP): ±80 V (positive & negative); TOF MS scan m/z range: 60–1,200 Da, Product ion scan m/z range: 25–1,200 Da, TOF MS scan accumulation time 0.25 s/spectra, Product ion scan accumulation time 0.03 s/spectra; The secondary mass spectrometry was obtained using Information Dependent Acquisition (IDA) and was in High Sensitivity mode, CE: 30 V ± 15. The blank growth control group was used to eliminate the interference of impurities on the instrument. The QC samples were used to test the stability of the instrument response and all samples were calibrated to reduce the error caused by the instrument.

The raw data were converted to the mzML format using ProteoWizard software (Mass spectrometry data format conversion tool) and processed with an in-house program, which was developed using R and based on XCMS, for peak detection, extraction, alignment, and integration. These raw data will be used for in-depth analysis of subsequent discussions. Then internal MS2 databases (Allwegene. DB) were used for metabolite annotation.

### 2.10 Statistical analyses

Data were analyzed using SPSS Statistics software (version 26.0) and expressed as mean ± standard error of the mean. Data differences between different groups were assessed by one-way analysis of variance (ANOVA) followed by Duncan’s *post hoc* test. Results with *p* values of <0.05 were considered statistically significant. Histograms and line charts were drawn with Origin 2022. Aipathwell (Fluorescence analysis software, Servicebio, Wuhan, China) was used to quantitatively analyze the fluorescence intensity in a specific region.

## 3 Results

### 3.1 Screening and LC-MS/MS analysis of antibacterial flavonoids in EtOAc extract of *L. gracile*

**FIGURE 1:**
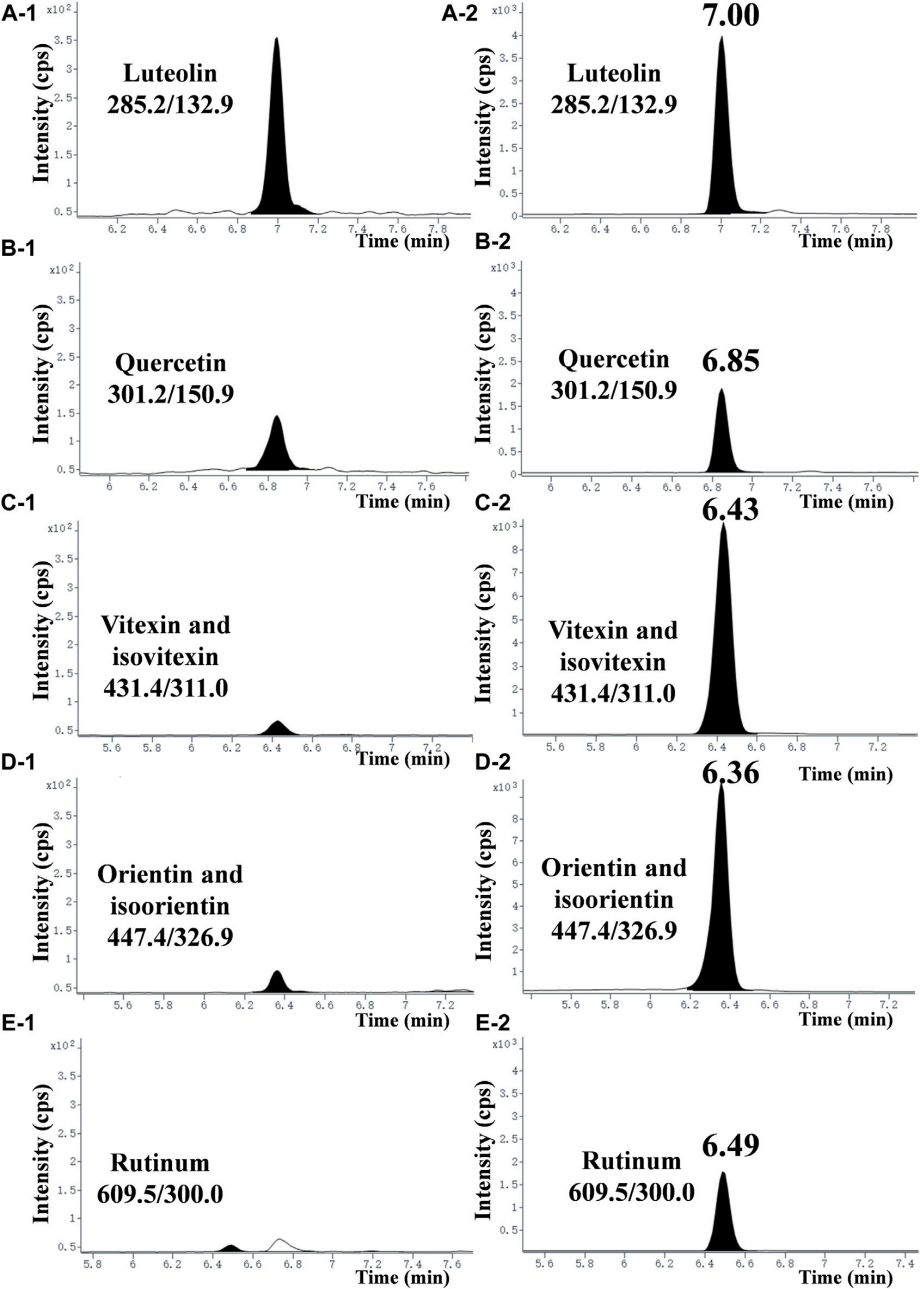
Typical chromatograms of seven flavonoids in the EtOAc extract. **(A-1)**, **(B-1)**, **(C-1)**, **(D-1)**, and **(E−1)** represent chromatograms of each compound at a concentration of 2.5 mg/mL in the EtOAc extract. **(A-2)**, **(B-2)**, **(C-2)**, **(D-2)**, and **(E−2)** represent chromatograms of each compound standard at a concentration of 100 ng/mL (purity >99.8%).

To explore the main antibacterial flavonoids of *L. gracile*, it was tracked and structurally identified by LC-MS/MS and broth dilution analysis. As shown in Table 1, the main antibacterial active site was the moderately polar EtOAc fraction (MIC = 12.8 mg/mL), indicating that the antibacterial active components were mainly concentrated in the EtOAc fraction. The antibacterial effect of the identified compounds was determined by the micro-broth dilution method to screen out the best active compound. The results of compound structure formulas (Supplementary Figure S1) and LC-MS/MS analysis (Figure 1) showed that the proportion of luteolin content in EtOAc extract was 3.43%, followed by 0.285% of quercetin. The proportion of rutin content was only 0.268%. Since orientin and isoorientin are isomers, the total proportion of content in the EtOAc extract was 0.144% after quantitative analysis. Similarly, vitexin and isovitexin are also isomers, with a total content of 0.107%. Meanwhile, most flavonoids in *L. gracile* have good antibacterial effects on *E. coli* standard and clinical MDR *E. coli*, and the MIC and MBC of luteolin on the tested bacteria were 1.0 mg/mL and 2.0 mg/mL, respectively.

**TABLE 1:** MICs and MBCs of different extract fraction of *Lophatherum gracile* and seven main ingredients in ethyl acetate fraction against MDR *Escherichia coli* (mg/mL).

	Monoblocs of *L. gracile* ethanol fraction	MDREC		*E. coli* (ATCC25922)	
		MICs	MBCs	MICs	MBCs
1	Petroleum ether extract	No effect	No effect	No effect	No effect
2	Ethyl acetate extract	12.8	12.8	12.8	12.8
3	N-butanol extract	No effect	No effect	No effect	No effect
4	Dichloromethane extract	25.6	25.6	25.6	25.6
5	Orientin	2	4	2	4
6	Isoorientin	4	8	4	8
7	Vitexin	4	8	4	8
8	Isovitexin	No effect	No effect	No effect	No effect
9	Rutinum	3.2	6.4	3.2	6.4
10	Luteolin	1	2	1	2
11	Quercetin	1	2	1	2

### 3.2 *In vitro* antibacterial activity of luteolin against MDR *Escherichia coli*

#### 3.2.1 Time-kill analysis

**FIGURE 2:**
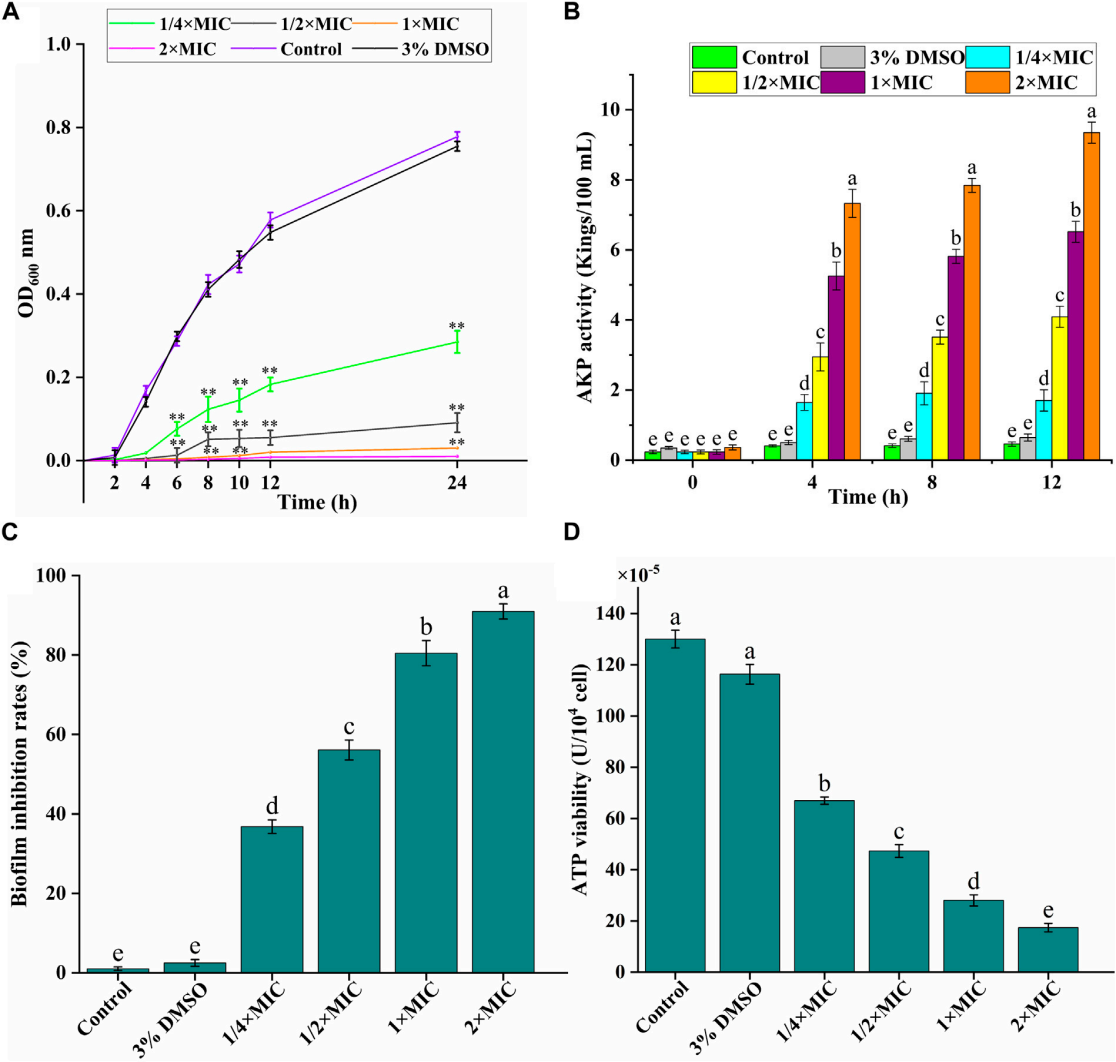
*In vitro* antibacterial activity of luteolin against multidrug-resistant *Escherichia coli* (MDR *E. coli*). **(A)** Effects of luteolin on bacterial cell growth. **(B)** Effects of luteolin on cell wall integrity, represented by the alkaline phosphatase (AKP) levels in the bacterial lysates. **(C)** Effects of luteolin on MDR *E. coli* biofilm formation was observed at OD_595_ nm. **(D)** Effect of luteolin on intracellular ATPase activity of MDR *E. coli.* All graphs show the mean ± SEM. *p < 0.05, **p < 0.01. Different lowercase letters on the bargraphs **(B,D)** indicate significant differences among treatments (one-way ANOVA, *p* < 0.05). The data are representative from three independent experiments.

As given in Figure 2A, the experimental group could significantly inhibit the growth of bacteria in a dose-dependent manner. Luteolin exhibited excellent antimicrobial activity at 1/4 × MIC 1/2 × MIC and MIC, and then had the bactericidal effect at 2 × MIC. The growth of the test bacteria in the control group and the 3% DMSO solvent control group was not significantly different and tended to be relatively stable.

#### 3.2.2 Leakage of intracellular content

AKP is an important index to evaluate the degree of cell wall damage. In this study (Figure 2B), the extracellular AKP level of the control group was maintained at about 0.5 Kings/100 mL, and the AKP release level of the experimental group increased significantly (*p* < 0.01) with the time and luteolin concentration, especially the 2 × MIC group.

#### 3.2.3 Evaluation of biofilm formation inhibition

We analyzed the effect of luteolin on the inhibition of the biofilm formation ability of the MDR *E. coli*. As exhibited in Figure 2C, the inhibition of luteolin on the biofilm formation of the MDR *E. coli* increased significantly (*p* < 0.01) with the increase of concentration, in a dose-dependent manner. The inhibition rates of biofilm formation of MDR *E. coli* were 80.45% and 87.98%, after being treated with luteolin at 1 × MIC, and 2 × MIC for 16 h, respectively.

#### 3.2.4 Evaluation of bacterial energy weakening

ATP is essential in the life activities of bacteria. In our study (Figure 2D), compared with the blank growth control group, the ATP activity level in the bacteria decreased significantly (*p* < 0.01), and gradually decreased with the increase of luteolin concentration. The results showed that luteolin could inhibit the normal energy synthesis of the tested bacteria, thereby inhibiting the normal growth, reproduction and replication of the bacteria until death.

### 3.3 Morphological changes of MDR *Escherichia coli* induced by luteolin

**FIGURE 3:**
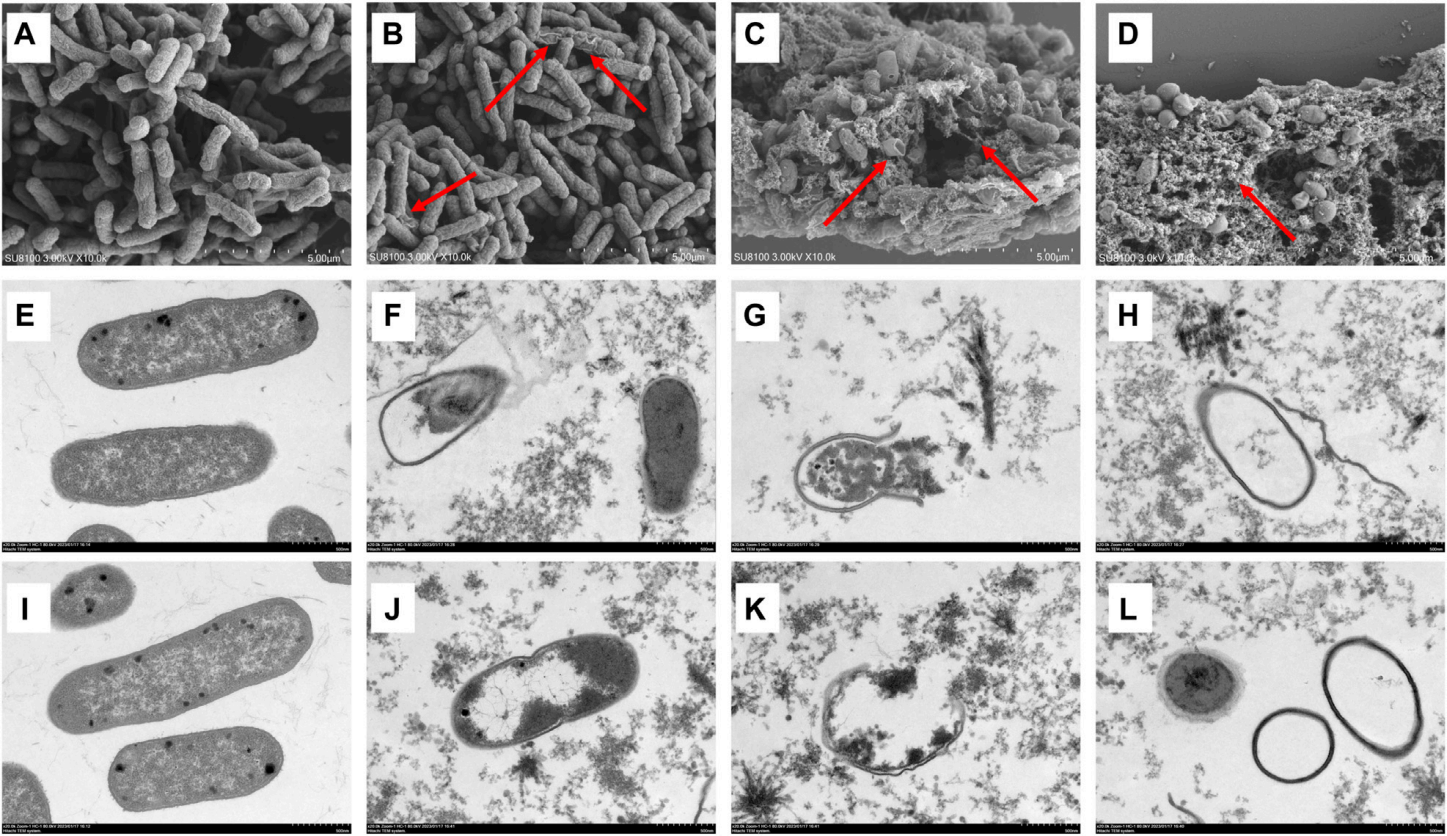
Effect of luteolin on the ultrastructure of multidrug-resistant *Escherichia coli* (MDR *E. coli*). SEM images of MDR *E. coli* (×20 k, **(A–D)**, without luteolin **(A)**, treated with 1/2 × MIC of luteolin **(B)**, 1 × MIC of luteolin **(C)** and 2 × MIC of luteolin **(D)**. TEM images of MDR *E. coli* (×20 k, E-L), without luteolin **(E,I)**, treated with 1 × MIC of luteolin **(F–H)**, 2 × MIC of luteolin **(J–L)**.

The morphological changes in the MDR *E. coli* before and after treatment with luteolin were observed using SEM and TEM. The results showed that the cell structure of the MDR *E. coli* in the control group was complete with a typical rod-like structure (Figure 3A); the ultrastructure of the bacteria was clear and complete, and the boundary of the inner and outer membranes of the cells was undamaged with distributed dense cytoplasm (Figures 3E, I). In SEM observation, the surface of a small number of bacteria treated with luteolin at 1/2 × MIC was rough and began to shrink (Figure 3B). However, the morphological structure of most bacteria treated with MIC of luteolin changed significantly (Figure 3C), mainly manifesting in swelling and shrinkage, cell deformation, and a large number of bacterial contents leakage. After exposure to the 2 × MIC of luteolin, almost no normal bacterial structure was observed (Figure 3D). TEM results showed that the ultrastructural damage of bacteria treated with MIC (Figures 3F–H) and 2 × MIC (Figures 3J–L) of luteolin exhibited a common plasmolysis phenomenon. The cellular content was reduced and unevenly distributed, and the cell wall/membrane was lysed with blurred, thinned, or even disappeared edges (Figures 3F, J). Cytoplasmic content leakage (Figures 3G, K) and the empty sac structure caused by the autolysis of intracellular organelles (Figures 3H, L) were observed. The damage of bacteria was exacerbated with the increase in luteolin dose. The results of TEM showed that luteolin had a targeting effect on different parts of bacteria, namely, the destruction of cell walls and cell membrane structural integrity.

### 3.4 Bacterial viability analysis

**FIGURE 4:**
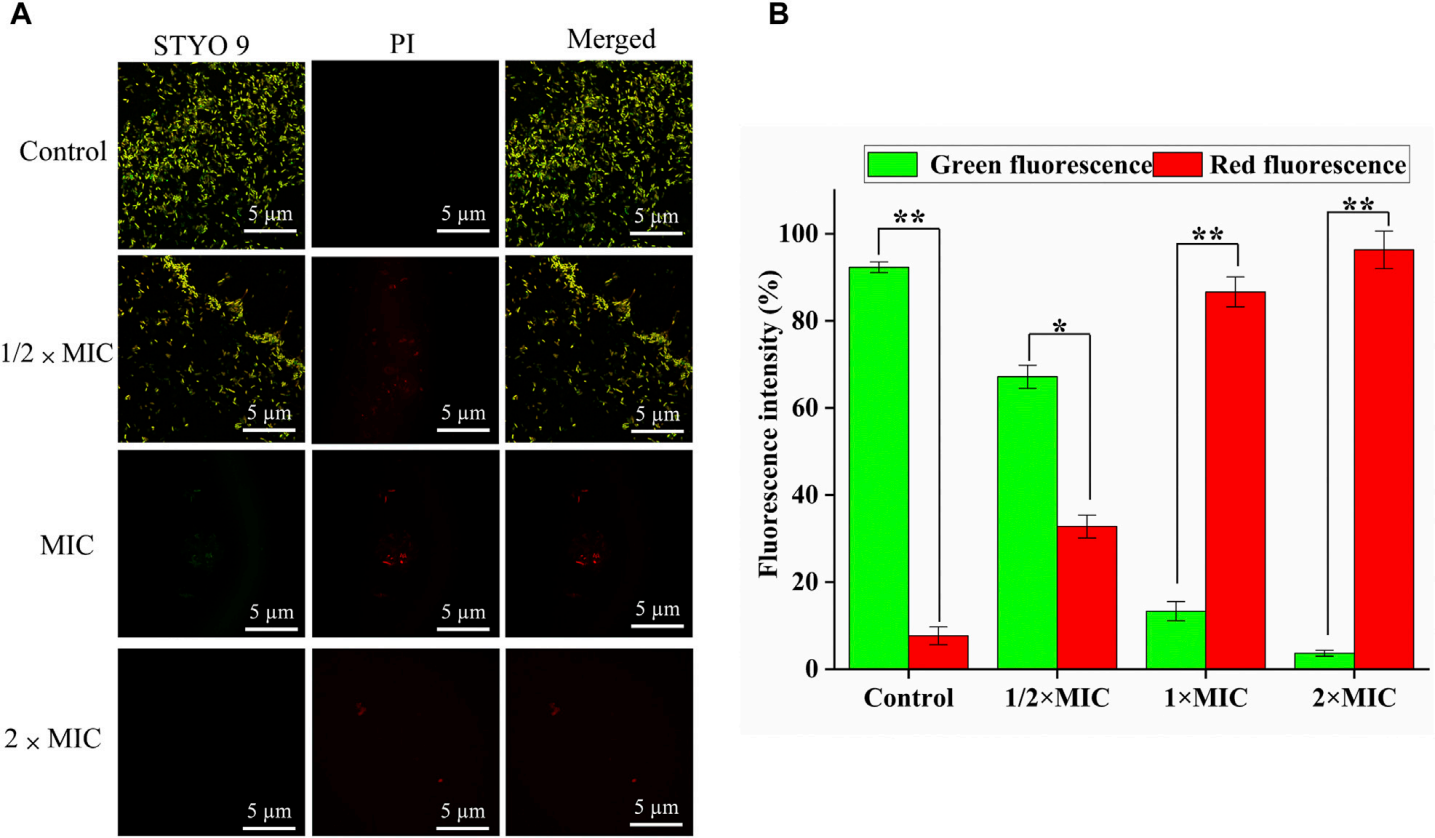
Bacterial viability assay. **(A)** CLSM images of multidrug-resistant *Escherichia coli* (MDR *E. coli*) with the treatment of luteolin and blank growth group with SYTO 9 (labels live bacteria) and PI (labels dead bacteria) (scale bar = 5 μm). **(B)** The number of red and green light bacteria cells was calculated and quantified by Aipathwell software; the graphs show the mean ± SEM. *p < 0.05, **p < 0.01.

Generally, SYTO nine can emit green fluorescence by binding nucleic acid in bacteria through bacterial cell membranes. In contrast, PI can only penetrate the damaged cell membrane and combine with nucleic acid to emit red fluorescence. Therefore, “alive” bacteria with intact membrane structures showed green fluorescence, and “dead” bacteria with damaged membrane structures showed red fluorescence for adjusting the appropriate ratio of SYTO 9 (2.5 μM) and PI (15 μM). The CLSM results of SYTO nine and PI staining are shown in Figure 4A, and the number of red and green light bacteria cells was calculated and quantified by Aipathwell software (Figure 4B). The MDR *E. coli* bacteria in the control group showed green fluorescence (almost no red fluorescence), whil as the luteolin concentration increased, the proportion of red fluorescence increased, indicating the sharp decrease (*p* < 0.01) of alive bacteria. Especially in the MIC and 2 × MIC of luteolin treatment groups, green fluorescence could hardly be observed.

### 3.5 Gene expression of drug resistance

**FIGURE 5:**
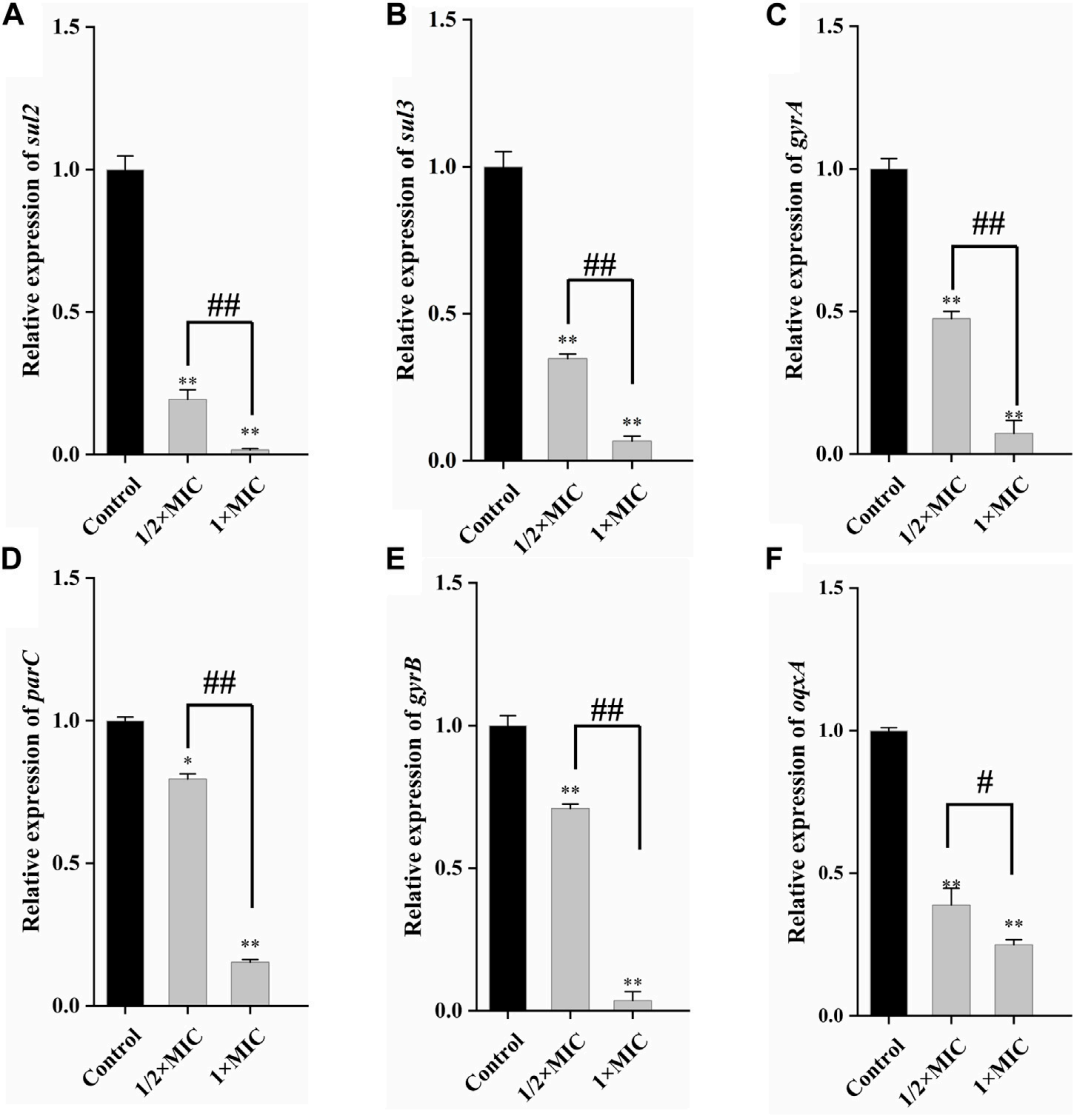
The effect of luteolin on the expression level of plasmid-mediated resistance genes in multidrug-resistant *Escherichia coli*. The graphs show the mean ± SEM. * or ^#^ *p* < 0.05, ** or ^##^ *p* < 0.01.

qRT-PCR could quantitatively determine the decrease of drug resistance genes carried by the tested strains, to better detect the weakening degree of luteolin on drug resistance genes. As given in Figures 5A, B, compared with the control group, 1 × MIC of luteolin highly (*p* < 0.01) downregulated the mRNA expression of *sul2* and *sul3*, and the inhibition rates were as high as 98.37% and 93.36%. Similarly, luteolin also significantly (*p* < 0.01) downregulated the expression levels of four drug resistance genes *gyrA*, *parC*, *gyrB* and oqxA, with inhibition rates of 92.77%, 96.43%, 84.7% and 75.04%, respectively (Figure 5C–F).

### 3.6 *In vitro* antibacterial mechanism of luteolin against MDR *Escherichia coli*

#### 3.6.1 Metabolomic analysis

**FIGURE 6:**
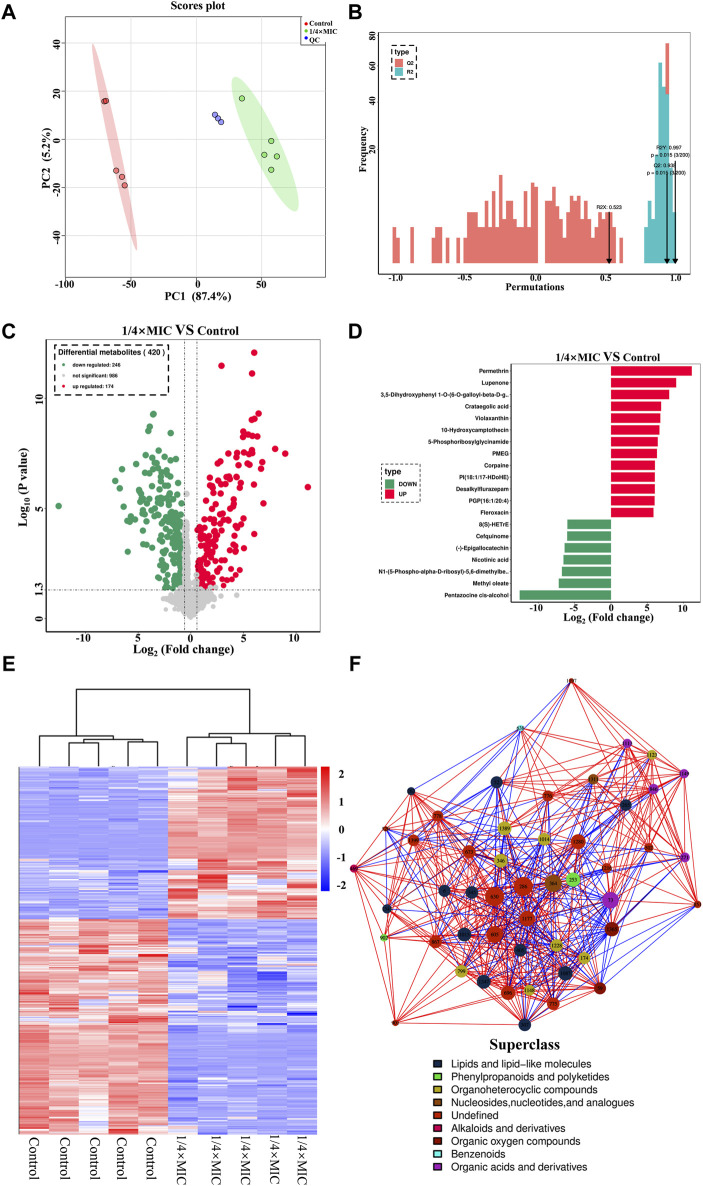
The PCA score scatter plot of all samples (including QC samples) **(A)**. The OPLS-DA model validation diagram of the combination was compared. In general, the model was best when *p* < 0.05 **(B)**. The multidrug-resistant *Escherichia coli* exposed to 1/4 × MIC luteolin and control. Volcanic map analysis differential metabolites **(C)**. Cluster heatmap analysis of differential metabolites **(D)**. Differential metabolites multiple histogram **(E)**. Differential metabolites network diagram **(F)**.

In the effect of luteolin on the growth curve of the MDR *E. coli*, it was found that 1/4 × MIC luteolin could significantly inhibit the growth rate of MDR *E. coli*. Therefore, 1/4 × MIC of luteolin was selected to treat MDR *E. coli* for 10 h for non-targeted metabolic analysis under the condition of capturing more metabolites. In the data processing of metabolites, 1,406 metabolites were identified by the combination of positive and negative ions. The PCA model undifferentiated analysis showed that the principal component PC1 variable can explain 87.4% of the original data (Figure 6A). The QC sample aggregation ensures the stability of the system and the precision of the instrument. There was no cross-distribution between the two groups, indicating that the difference between the control group and the treatment group was statistically significant. It also shows that the intracellular metabolic level of the MDR *E. coli* has changed significantly after luteolin treatment. Further to sort out the relationship between metabolites and sample categories, a supervised OPLS-DA model was constructed and the model parameters (R_2_ and Q_2_) were tested by permutation test (Figure 6B). The results showed that the R_2_Y and Q_2_ values were 0.997 and 0.938, respectively, indicating that the model had good interpretability and predictive ability, that is, the model was well constructed. The metabolites were visualized in the form of a volcano plot (Figure 6C). The results showed that 246 metabolites were significantly downregulated and 174 metabolites were significantly upregulated (*p* < 0.05). The normalized cluster analysis (Figure 6D) of the selected differential metabolites revealed that the differential metabolites were significantly variable and well segregated in the control group and the 1/4 × MIC luteolin group. The top 20 metabolites with the greatest influence on the antibacterial mechanism were screened out and analyzed by taking the difference multiple of metabolites between groups as the logarithm of the base number of 2 (Figure 6E).

Finally, to more intuitively reveal the co-regulation relationship between various metabolites, the correlation matrix is converted into a network diagram as shown in Figure 6F. The most influential metabolites in the whole network diagram were mainly derivatives of various metabolites (such as 8-C-p-Hydroxybenzylkaempferol, 5-Phosphoribosylglycinamide, Pantetheine 4-phosphate) followed by lipids and lipid-like molecules (such as glycerophospholipids and fatty amides), organic oxygen compounds (such as sugars and alcohols), organic acids and derivatives (such as amino acids and phospholipids), nucleosides, nucleotides, and analogs (such as amines). The variety of metabolites is the response of bacteria to changes in the external environment, and the increase or decrease of metabolites will affect the normal development and reproduction of MDR *E. coli*.

#### 3.6.2 Pathway analysis

**FIGURE 7:**
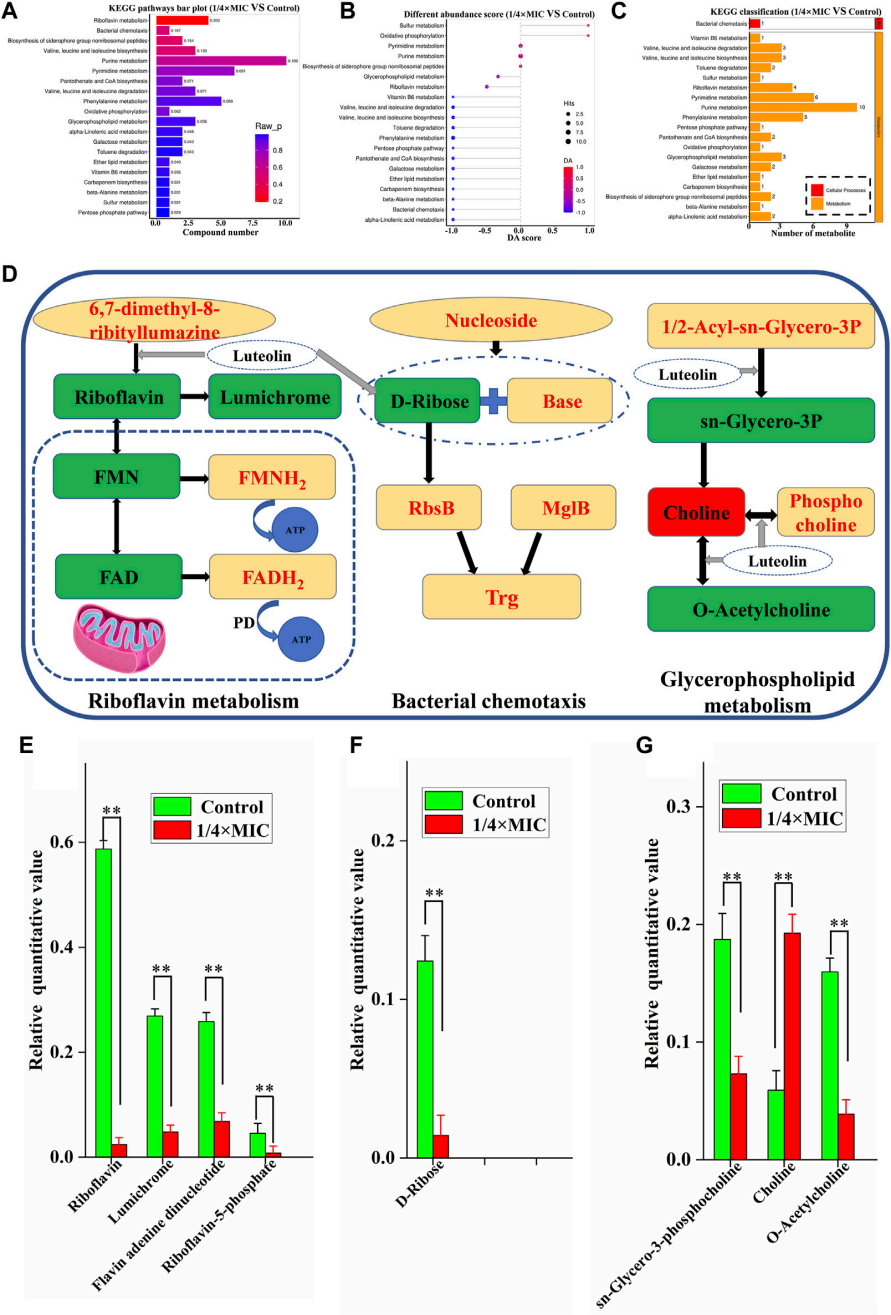
Histogram indicate the differential metabolic compound pathway enrichment analysis **(A)**. Differential abundance score plot of differential metabolic pathways, the depth of the color of the line segment and the dot is proportional to the DA score value, red indicates that the pathway is upregulated and blue indicates downregulated **(B)**. Metabolic pathway classification histogram **(C)**. The 1/4 × MIC of luteolin group in **(D)** had higher levels of metabolic compounds represented in red and lower levels of metabolic compounds represented in green compared to the control. Changes in identified metabolite biomarkers in response to luteolin exposure in the pathways of riboflavin metabolism, bacterial chemotaxis and glycerophospholipid metabolism in MDREC, *p < 0.05, **p < 0.01 **(E,F,G)**.

The pathways of differential metabolites were evaluated and classified by KEGG (http://www.kegg.jp). A total of 20 metabolic pathways with the most significant enrichment (*p*-value) were screened after treatment of MDR *E. coli* with 1/4 × MIC luteolin (Figure 7A). Then the differential abundance score evaluation found that the overall metabolic level of bacterial cells was downregulated (Figure 7B). Finally, the selected metabolic pathways were classified and analyzed (Figure 7C). The results showed that the metabolic pathways were roughly divided into two categories: metabolic and cellular processes. Therefore, the comprehensive analysis revealed that the changes in metabolites after luteolin intervention were highly correlated with the interference of riboflavin metabolism, bacterial chemotaxis and glycerophospholipid metabolism in the MDR *E. coli*.

In our analysis, riboflavin metabolism is the most relevant metabolic pathway affected by luteolin treatment (Figures 7D, E). Compared with the control group, the metabolic levels of riboflavin, lumichrome, flavin adenine dinucleotide (FAD) and flavin mononucleotide (FMN) in the pathway were significantly decreased in the 1/4 × MIC luteolin treatment group (*p* < 0.01). Riboflavin (also known as vitamin B_2_) is the precursor of FAD and FMN. Besides, the relative level of D-Ribose in the MDR *E. coli* cell pathway (Bacterial chemotaxis) was significantly (*p* < 0.01) decreased under the intervention of luteolin (Figures 7D, F). Moreover, luteolin destroyed the glycerophospholipid metabolic pathway of MDR *E. coli* (Figures 7D, G). The results showed that compared with the control group, the levels of sn-Glycero-3-phosphocholine and O-Acetylcholine metabolites in this pathway were significantly decreased (*p* < 0.01), while the levels of Choline metabolites were significantly increased (*p* < 0.01).

## 4 Discussion

Compared with antibiotic-sensitive strains, infections caused by drug-resistant *E. coli* are more difficult to treat, resulting in higher mortality and treatment costs. The WHO has predicted that by 2050, the number of global deaths caused by antibiotic resistance will increase from 700,000 to about 10 million per year (de Kraker et al., 2016). *E. coli* can obtain drug resistance by capturing exogenous genes/chromosomal mutations and transmitting drug resistance genes horizontally (Poirel et al., 2018). Faced with such a serious problem of drug resistance, many researchers have focused on the development of natural botanicals from plant extracts. Notably, unlike traditional antibiotics with specific targets, natural botanicals have multiple targets and exhibit a variety of antibacterial molecular mechanisms, so the possibility of drug resistance is extremely low. Moreover, natural flavonoids have been shown to be inhibitors of a variety of enzyme structures, which help to enhance antibacterial activity. For example, flavonoids can competitively interact with binding pockets to inhibit the synthesis of DNA gyrase, thereby inhibiting the synthesis of nucleic acids and affecting the metabolism of drug-resistant bacteria to achieve antibacterial effects (Górniak et al., 2019).

Accordingly, these botanicals of flavonoids are the interest of researchers in many antibacterial studies (Jang et al., 2021). We also speculate that the highest content of flavonoid in *L. gracile* is the main antibacterial active ingredient. Therefore, the ethanol extract of *L. gracile* was divided into four parts with different polarities by site separation method, and the best active part of the antibacterial effect was screened by the macroscopic broth dilution method. The results showed that the EtOAc extract had the best antibacterial effect. Further separation and purification of the EtOAc extract helps to screen compounds with antibacterial activity. This study showed that the main components of the ethanol extract of *L. gracile* had seven flavonoids. The structures and contents of antibacterial active ingredients in the EtOAc fraction were identified and quantified based on the above-mentioned literature and comparison with known standard substances by LC-MS/MS analysis. The results showed that the antibacterial effect of luteolin is better than other compounds except quercetin. However, the content of quercetin is quite low, so the main antibacterial ingredient in *L. gracile* is luteolin, indicating that luteolin has more advantages in the treatment of clinical drug-resistant strains of *E. coli.* Therefore, it is more meaningful and valuable to study the antibacterial mechanism of luteolin.

The time-killing curve experiment can observe the inhibitory effect of luteolin on MDR *E. coli*, and determine whether the killing effect of the compound is dose-dependent or time-dependent and further determine whether the compound has bactericidal or bacteriostatic effects (Zhu et al., 2021). The study found that luteolin showed bactericidal effect at high concentrations and antibacterial effect at low concentrations in a dose-dependent manner. To assess the integrity of the cell wall, we measured AKP leakage. Under normal conditions, AKP only exists in the cell and cannot be detected *in vitro* (Deng et al., 2021). The higher the level, the higher the degree of cell wall damage (Zhao et al., 2018). In our study, luteolin could significantly cause the leakage of AKP in the cells of the tested bacteria in a dose and time-dependent manner, resulting in the inability of bacterial cells to withstand changes in osmotic pressure and cell body deformation or cleavage, suggesting that luteolin could lead to cell death by destroying the cell wall of bacteria or increasing its permeability.

The destruction of the internal and external structures of MDR *E. coli* induced by luteolin was observed using SEM and TEM, and the destructive effect of luteolin on the tested bacteria was dose-dependent. Such uneven distribution and leakage of bacterial contents can lead to the growth inhibition or even death of bacteria (Shen, et al., 2015), which indicated that the damage to cell wall and cell membrane may be the reason for the observed growth inhibition and bactericidal effect. This is consistent with the previously reported pattern (He et al., 2021).

Besides, biofilm is a complex microbial community structure formed by the interaction between bacteria and covered by metabolites such as polysaccharides produced by themselves in the extracellular environment (Yu et al., 2018). The unique metabolic activity can produce an acidic environment to invalidate antibiotics, which makes the treatment of bacterial chronic infection more complicated (de Breij et al., 2018; Bhatia and Banerjee, 2020). Our results suggest that luteolin has a strong and sustainable clean-up effect on the biofilm formation of MDR *E. coli*, and can be used as a possible natural biofilm inhibitor to preserve food to inhibit the formation of biofilm on food surfaces.

Na^+^K^+^-ATPase widely exists in animal, plant and microbial cells, which can catalyze the hydrolysis of ATP to ADP and inorganic phosphorus (Jorgensen et al., 2003). Therefore, this experiment determined the change of ATP level by measuring the content of inorganic phosphorus. Through the determination of ATP level, it was found that luteolin induced the ATP synthesis of the tested bacteria to be blocked. This will lead to the synthesis of ATP in bacteria and energy intake, metabolism, storage and utilization of a variety of closely related life activities cannot be carried out normally. This result is consistent with previous studies (Cui et al., 2018).

The survival of MDR *E. coli* induced by luteolin was determined by adjusting the mixing ratio of SYTO 9 (2.5 μM) and PI (15 μM) dyes. (Robertson et al., 2019). This result indicates that luteolin could inhibit the viability of MDR *E. coli* by destroying the integrity of bacterial cell membranes. The higher the concentration of luteolin, the more effective it is to inhibit the growth of MDR *E. coli*. Furthermore, this also implies that luteolin may cause the disruption of gene transcription or expression, downregulation of drug resistance gene expression levels, or blocking protein synthesis (Zheng et al., 2023).

The increase in drug resistance has impaired the clinical efficacy of sulfonamides and seriously threatened global public health (Venkatesan et al., 2023). The clinically isolated MDR *E. coli* is resistant to sulfonamides through gene replacement, that is, obtaining the dihydropteroate synthetase (DHPS) substitutes encoded by the drug resistance genes *sul2* and *sul3* (Zhou et al., 2021). In this mechanism, the expression product has a low affinity to sulfonamides. In this study, the induction of lignans could effectively inhibit the synthesis of DHPS in MDR *E. coli* and hinder the normal growth of bacteria. Therefore, we can achieve the purpose of weakening bacterial resistance or restoring the use of sulfonamides by adding a certain amount of luteolin.

In recent years, the significant increase in the resistance of *E. coli* to quinolones has been mainly due to mutations that reduce target affinity and drug accumulation (Jacoby, 2017). However, quinolones are still an important treatment option for a variety of clinical indications (Imkamp et al., 2023). Therefore, it is crucial to find natural antibacterial compounds to eliminate the resistance of quinolones. Generally, the *gyrA* and *parC* genes carried by the MDR *E. coli* strains could change the amino acid sites encoding gyrase or topoisomerase IV, leading to quinolone resistance. The *gyrB* gene mediates drug resistance through mutations in acidic residues in the quinolone resistance determining region (QRDR). A report has found that mutations in the QRDRs of these three genes seemed to work by reducing the affinity of quinolones to enzyme-DNA complexes (Hooper and Jacoby, 2015). Luteolin highly downregulated the mRNA expression of these three genes (*parC*, *gyrA*, *gyrB*) by qRT-PCR, which could exert antimicrobial effects by recovering the affinity of quinolones to the enzyme-DNA complex. In addition, the *oqxA* gene is an efflux pump gene in the RND family. It could encode a membrane fusion efflux pump protein, which can embed in the cell membrane and expel fluoroquinolones from the bacterial cytoplasm to reduce the drug concentration in the body and produce low drug resistance cytoplasm (Yanat, et al., 2017). The downregulation of *oqxA* mRNA expression level revealed that luteolin could inhibit the expression of efflux pump protein to weaken MDR *E. coli* resistance.

In the intracellular metabolic pathway, significant changes in certain key metabolites can prevent the normal growth and reproduction of bacteria, leading to bacterial lysis or death. Untargeted metabolomics provides key metabolites and metabolic pathways for the direct death of bacteria. The bacterial cell biochemical network diagram systematically explains how bacteria respond to changes in the external environment and reveals the detailed process of the antibacterial mechanism (Zhu et al., 2018). Luteolin is a polyphenolic compound, which is converted into acidic or alkaline compounds (such as lupenone, crataegolic acid, 10-hydroxycamptothecin) under the action of various digestive enzymes in MDR *E. coli* cells. It prevents the metabolic process of bacteria from multiple pathways, leading to the increase of amino acids, amides, esters and other substances, and ultimately accelerates the death of bacteria (Gaur and Gazle, 2023; Velderrain-Rodríguez et al., 2014).

The significant changes in vitamins, amino acids, nucleotides, and phospholipids are usually important indicators for determining the cessation of growth and reproduction of bacteria after interference during the metabolic process of bacteria. Among them, the riboflavin metabolic process is the most critical pathway that affects the metabolic level of MDR *in vivo* induced by luteolin. Riboflavin (also known as vitamin B_2_) is the precursor of FAD and FMN. Moreover, riboflavin is also a cofactor of flavin protein (García-Angulo, 2017). However, lack or low levels of riboflavin, FAD and FMN could lead to the accumulation and inactivation of flavin proteins (Sebastián et al., 2018). Therefore, the inactivation of flavin protein will not be able to perform flavin-dependent basic functions (such as electron transport in the respiratory chain and photosynthetic chain, β-oxidation of fatty acids, and nucleotide synthesis or signal transduction) to inhibit ATP synthesis and DNA replication in bacterial cells, and ultimately accelerate bacterial death, which is corresponding to the significant downregulation of the MDR *E. coli* multidrug resistance genes. In addition, lumichrome is a derivative of riboflavin, and the decrease in its metabolic level may inhibit the signal transduction of the bacterial quorum sensing system and lead to the inhibition of bacterial growth (Rajamani et al., 2008). Since 6,7-dimethyl-8-ribityllumazine is a direct precursor of riboflavin synthesis, riboflavin is produced by riboflavin synthase-mediated disproportionation reaction. Therefore, riboflavin synthase may be a potential new target of luteolin against MDR *E. coli*, that is, to inhibit riboflavin metabolism from the beginning.

Bacterial chemotaxis is considered to be an important cellular process involved in bacterial pathogenicity, symbiosis and biofilm formation, maintaining bacteria in their optimal environmental niche, which is likely to be the cause of bacterial resistance (Wadhams and Armitage, 2004). Interestingly, RbsB is a functional homologue of d-ribose-binding periplasmic proteins in *E. coli*. The low levels of D-Ribose prevent RbsB from binding to chemoattractant protein III (trg), limiting the movement of bacteria to sites suitable for growth and ultimately leading to growth inhibition (Rico et al., 2016). Therefore, luteolin inhibits bacterial chemotaxis by inhibiting the synthesis of D-Ribose to reverse the MDR *E. coli* resistance.

It has been reported that Choline can provide glycine as the sole carbon source for bacterial growth through glycine betaine conversion (Wargo, 2013). Bacteria have components similar to the nervous system of mammals, which can secrete neurotransmitters to protect themselves by making an immune response to the external environment, such as O-Acetylcholine (Islas Weinstein et al., 2015). However, O-Acetylcholine biosynthesis is reversible. Luteolin may promote the reverse synthesis of Choline by sn-Glycero-3-phosphocholine and O-Acetylcholine. The inability of Choline to convert leads to the inability of the MDR *E. coli* to utilize carbon sources and to respond to the external environment, which ultimately leads to the cessation of growth.

Based on these findings, we first sorted out and predicted the preliminary phenotype of the antibacterial effect and the changes of internal metabolites to explain the antibacterial mechanism and target of the natural polyphenol compound luteolin. This study also shows that luteolin is not determined by a specific antibacterial mechanism like other commonly used antibiotics, but achieves antibacterial effects through multiple action targets and modes of action. Therefore, from the perspective of bacteria, compared with clinically used antibiotics, the possibility of drug resistance to luteolin is extremely low or the time leading to drug resistance is very long. Comprehensive analysis, the antibacterial mechanism of luteolin against the tested bacteria is to first significantly destroy the structural integrity of the cell membrane/wall of the tested bacteria, increasing membrane/wall permeability, and thereby accelerating the entry of luteolin into the bacteria. Then, luteolin continued to destroy the structure of bacterial cells to make their inner concave shrinkage, swelling and degeneration, and plasmolysis, resulting in a large leakage of bacterial cytoplasm and the inability to carry out the normal life activities of bacteria. Finally, luteolin significantly affected the normal metabolic process of bacteria, hinders the synthesis of ATP, and made bacteria unable to carry out the next step of cell membrane/wall synthesis, resulting in the normal replication and reproduction of bacteria, to achieve the effect of antibacterial or reducing its drug resistance.

## 5 Conclusion

In summary, *L. gracile*. is a natural antibacterial product. The EtOAc fraction with strong antibacterial activity was isolated from the ethanol extract of *L. gracile*. After the quantification of the main antibacterial active compounds by LC-MS/MS, luteolin was proven to exhibit excellent antibacterial activity. The bacterial viability treated with luteolin decreased in a dose-dependent manner. Luteolin could damage the integrity of the cell wall and membrane of bacteria, resulting in significant morphology changes in the bacterial surface. Such changes can increase the permeability of the MDR *E. coli* and the leakage of intracellular substances, leading to the lysis and death of bacteria cells. More importantly, luteolin significantly downregulated the expression levels of MDR *E. coli* resistance genes and multiple metabolic pathways, resulting in a decrease in ATP synthesis. This further proves that luteolin could weaken or reverse bacterial resistance by molecular mechanisms. This work will benefit in finding natural antibacterial compounds to prevent and treat multidrug-resistant bacterial infections, and also shows great potential in the development of alternatives to antimicrobial agents.

## Data Availability

The original contributions presented in the study are included in the article/Supplementary Material, further inquiries can be directed to the corresponding authors.
