# Supplementary material for: Antibacterial activity and mechanism of luteolin isolated from Lophatherum gracile Brongn. against multidrug-resistant Escherichia coli
Source: Front Pharmacol. 2024 Jun 24;15:1430564. doi: 10.3389/fphar.2024.1430564 (PMC11232434; doi:10.3389/fphar.2024.1430564)
Supplement: Supplementary file 1 [file DataSheet1.DOCX]

***Supplementary Information***


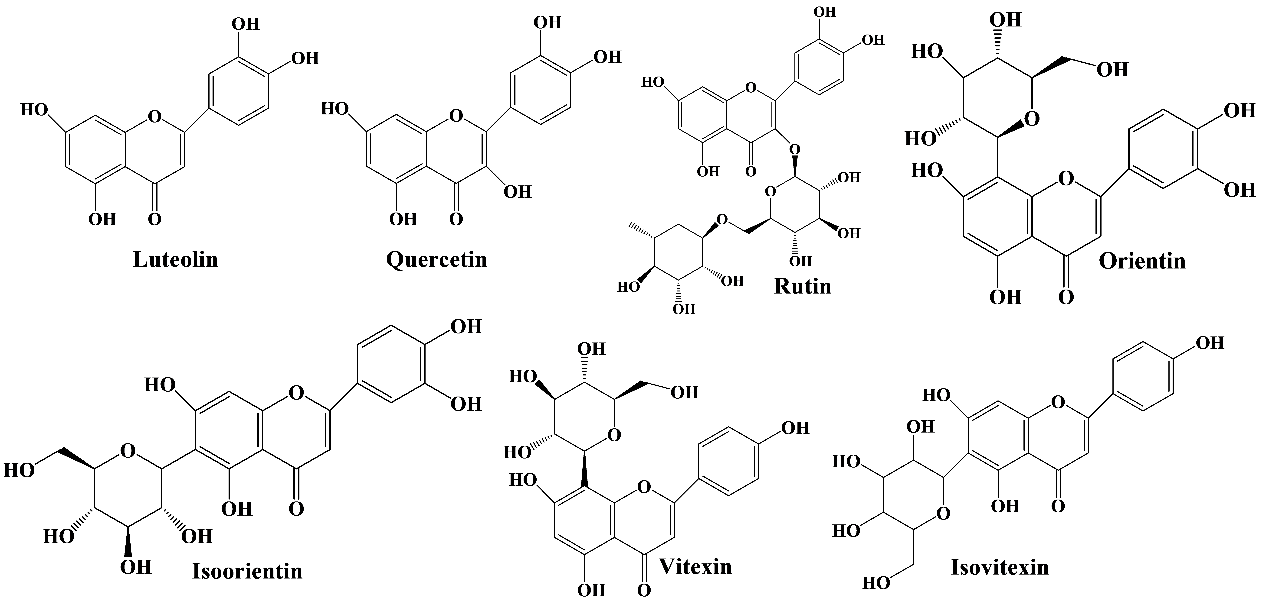


**Supplementary Figure 1.** Structural formulas of seven major flavonoids.

| **Table S1**  Mass spectral parameters for 7 flavonoids compounds in negative ion mode. | | | | | | |
| --- | --- | --- | --- | --- | --- | --- |
| Compounds | Precursor ion  (m/z) | Product ion  (m/z) | Dwell (s) | Fragmentor (V) | cell accelerator voltage | CE (V) |
| Orientin and Isoorientin | 447.4 | 357^*^ | 50 | 150 | 5 | 24 |
|  |  | 326.9 | 50 |  | 5 | 24 |
| Vitexin and Isvitexin | 431.4 | 311.0^*^ | 50 | 150 | 5 | 24 |
|  |  | 283 | 50 |  | 5 | 40 |
| Rutin | 609.5 | 300.0^*^ | 50 | 180 | 5 | 40 |
|  |  | 271 | 50 |  | 5 | 60 |
| Luteolin | 285.2 | 150.9^*^ | 50 | 120 | 5 | 28 |
|  |  | 132.9 | 50 |  | 5 | 40 |
| Quercetin | 301.2 | 178.9^*^ | 50 | 90 | 5 | 20 |
|  |  | 150.9 | 50 |  | 5 | 24 |
| Note: Product ion, the product ion (*) of each analyte was used for quantiﬁcation, and the other was used for identiﬁcation; CE, collision energy. | | | | | | |

| **Table S2**  Specific primer sequences for qRT-PCR | | | |
| --- | --- | --- | --- |
| Gene | Primers (5`-3`) | Target bands (bp) | Annealing (℃) |
| *sul-3* | Forward: ATCTCCGCACCCATGATGTTAAGTC  Reverse: CCAGACCTAAGTCAAGTACGCCAAC | 142 | 59 |
| *sul-2* | Forward: TGGCGACATCATGGATCACATTGC  Reverse: AGGACAAGGCGGTTGCGTTTG | 92 | 60.3 |
| *parC* | Forward: CATGCTGCCGTCTGAACCTGTC  Reverse: TCTTACCTTTCACCGCCGCTTTG | 137 | 61.5 |
| *gyrA* | Forward: CGAGTTCAACCGTCTGCGTACC  Reverse: ACCTTCAGCGGAGAACAGCATTAC | 121 | 61 |
| *gyrB* | Forward: CCATTCACGCCGATAACTCTGTCTC  Reverse: GATCACTTCCGCCGCCGATAC | 98 | 59.8 |
| *oqxA* | Forward: TGGCAGACGGTTTACGCATCG  Reverse: GCTGGTGGTCATGGCAACGG | 128 | 60.9 |
| *16SrRNA*  *(Reference gene)* | Forward: AACTGGAGGAAGGTGGGGATGAC  Reverse: CGGACTACGACGCACTTTATGAGG | 135 | 60 |
